# Astragaloside IV inhibits palmitic acid-induced apoptosis through regulation of calcium homeostasis in mice podocytes

**DOI:** 10.1007/s11033-021-06204-4

**Published:** 2021-02-19

**Authors:** Yingjun Zang, Shuang Liu, Aili Cao, Xiangyu Shan, Wenjuan Deng, Zhijun Li, Hao Wang, Yunman Wang, Li Wang, Wen Peng

**Affiliations:** 1 Department of Nephrology, Putuo Hospital, Shanghai University of Traditional Chinese Medicine, Shanghai, 200062 People’s Republic of China; 2 Laboratory of Renal Disease, Putuo Hospital, Shanghai University of Traditional Chinese Medicine, 164 LanXi Road, Shanghai, 200062 People’s Republic of China

**Keywords:** Astragaloside IV, Podocyte apoptosis, Calcium homeostasis, Endoplasmic reticulum stress, TRPC6

## Abstract

Loss of podocytes is a hallmark of diabetic nephropathy, and a growing body of evidence indicates that podocytes are susceptible to palmitic acid (PA). We have previously shown that AS-IV inhibited PA-induced podocyte apoptosis by activating sarcoendoplasmic reticulum Ca^2+^ ATPase (SERCA), which indicate calcium regulation may involve in the process. Immunofluorescence staining, Western blot and flow cytometry were used to measure the protective efficacy of AS-IV to ameliorate PA-induced ER stress and podocyte apoptosis. Meanwhile, AS-IV inhibited cytochrome c release, decreased mitochondrial membrane potential, accompany with the depletion of endoplasmic reticulum Ca^2+^ and elevation of cytosolic and mitochondrial Ca^2+^. Sequestration of cytosolic calcium with BAPTA-AM limited the response of podocyte apoptosis, while during the process the effect of AS-IV was also restrained. In contrast, elevation of cytosolic calcium with calcium ionophore ionomycin was depressed by AS-IV addition. Furthermore, inhibiting TRPC6 expression with SKF96365 or TRPC6 siRNA counteracted the beneficial effect of AS-IV. Our study provides further evidence to conclude the inhibitory effect of AS-IV to podocyte apoptosis is Ca^2+^-dependent. And the efficacy correlates with inhibiting TRPC6-mediated Ca^2+^ influx, and then cellular Ca^2+^ disturbance was coordinated.

**Supplementary Information:**

The online version contains supplementary material available at 10.1007/s11033-021-06204-4.

## Introduction

Podocytes are terminally differentiated epithelial cells in the filtration barrier of the kidney. Loss of podocyte functions, especially podocyte apoptosis, is considered as an early and key event in the development of glomerulosclerosis that leads to diabetic nephropathy (DN) and other end-stage renal disease eventually [1, 2]. One of the major pathogenic mediators in type 2 diabetes and its complications is dyslipidemia, representative with high saturated free fatty-acid (FFA) concentrations in blood [3]. During the process, saturated FFA palmitate is one of the proapoptotic factors that result to podocyte endoplasmic reticulum (ER) Ca^2+^ depletion, following with alteration of cytosolic and mitochondrial matrix Ca^2+^ concentrations, and ultimate apoptosis [4, 5]. Regulation of Ca^2+^ homeostasis is essential for protecting against podocyte injury, and inhibiting the development of diabetic complications.

It is universally recognized that extracellular stimuli increase intracellular Ca^2+^ levels through either promoting its release from intracellular organelles or its entry across the plasma membrane. The class C transient receptor potential (TRPC) channels are Ca^2+^-permeable cation channels expressed in the plasma membrane of a large amount of tissue and cell type including kidney [6]. TRPC6, which is one of the members of TRPC family channels that expressed in podocyte and represents a component of the glomerular slit diaphragm [7]. What is noteworthy is that Ca^2+^ entry via TRPC6 can lead to intracellular Ca^2+^ release, ultimately resulting in podocyte ER stress, mitochondrial dysfunction, cytoskeleton rearrangement and apoptosis. Palmitic acid, high glucose and albumin overload contribute to those processes of abnormalities [5, 8, 9]. Thus, pharmacological targeting of TRPC6 signaling pathways in mediating Ca^2+^ signaling is considered as a developing direction for treatment of DN.

Astragaloside IV (AS-IV) is a representative saponin isolated from *Astragalus membranaceus (Fisch) Bge*, which possesses various pharmacological activities such as inhibiting fibrosis, oxidative stress and apoptosis in kidney disease [10–12]. Our previous investigation showed that AS-IV treatment could ameliorate podocyte injury via sarcoendoplasmic reticulum Ca^2+^ ATPase 2 (SERCA2)-dependent ER stress reduction, which indicates that Ca^2+^ may take part in the regulatory role of AS-IV on podocyte apoptosis [13, 14]. In this study, we further showed that Ca^2+^ homeostasis was disrupted with the administration of palmitic acid (PA), accompanied with the ascending cytochrome c release and mitochondrial membrane potential. While AS-IV treatment improved these perturbations of cellular homeostasis. Thus, the efficacy of AS-IV linking with PA-induced ER stress and apoptosis in mouse podocyte was determined, and the mechanism relative to TRPC6 was also been investigated.

## Material and methods

### Materials

Astragaloside IV (purity at 98%) was purchased from Shanghai Bogoo Biotechnology Company (Shanghai Co. Ltd., China). Palmitic acid, BAPTA-AM and SKF96365 were obtained from Sigma–Aldrich (St. Louis, MO, USA). Mag-Fura-2, Rhod-2 and JC-1 were purchased from Invitrogen (Carlsbad, CA, USA). Fluo-4 was purchased from Dojindo (Kumamoto, Japan) and OAG was purchased from MedChem Express (Monmouth Junction, NJ, USA).

### Cell culture

Immortalized mouse podocyte cell lines originally were verified by Dr. Peter Mundel (Division of Nephrology, Massachusetts General Hospital, Harvard University) and kindly donated by Prof. Niansong Wang (Shanghai Sixth People’s Hospital, China). Podocytes were grown on type 1 collagen (BD Bioscience, Bedford, MA) at 33°C in the presence of 10 U/mL mouse recombinant interferon-γ (Invitrogen, Carlsbad, CA), 10% heat-inactivated fetal calf serum (Biochrom, Ltd., Cambridge, UK), 100 U/mL penicillin and 100 μg/mL streptomycin in RMPI 1640 for proliferation. To induce differentiation, podocytes were maintained at 37 °C without IFN-γ for 14 days. Previous study found that palmitic acid concentration at 250 µM is suitable to induce podocyte apoptosis, so the preparation was continued as described [5]. Briefly, a 20 mmol/L solution of palmitic acid in 0.01 mol/L NaOH was complexed to 5% BSA in a molar ratio of 8:1. Then the mixture was sterile filtrated and added to culture medium to achieve the final palmitic acid concentration of 250 µM in the medium. The final concentration was measured using a commercial kit (WakoChemicals, Richmond, VA, USA).

Cells were growth arrested in RMPI-1640 containing 1% serum before being pretreated with or without AS-IV (10, 20, 40 or 80 μM) for 12 h followed by treatment with BSA or 250 μM palmitate acid for 24 h. AS-IV was dissolved in DMSO and the final DMSO concentration did not exceed 0.1% (v/v).

### Staining for endoplasmic reticulum and mitochondria

Cells were seeded at a concentration of 2.5 × 10^5^ cells into 6-well plates for 24 h. Podocytes were incubated with palmitic acid in the presence or absence of AS-IV for the indicated concentrations.

To confirm the endoplasmic reticulum (ER) localization, cells were loaded with ER-tracker (Cayman Chemical, Ann Arbor, MI, USA) in HBSS for 15 min at 37 °C, washed with HBSS and incubated with Hoechst 33342 (Thermo Fisher Scientific, Waltham, MA, USA) to nuclei staining for 3 min, washed with PBS, and images were taken using a LEICA laser scan microscope (LEICA DM IRB, Leica, Wetzlar, Germany).

To confirm the ER localization of the Mag-fura2 probe, cells were loaded with ER-tracker in HBSS for 30 min at 37 °C, washed with HBSS and incubated with Mag-fura2 for another 30 min at 37°C. The cells were then washed with HBSS (without Ca^2+^) and incubated with Hoechst 33342 to nuclei staining for 5 min, washed with HBSS (without Ca^2+^) again and visualized by the fluorescence microscopy using LEICA laser scan microscope.

To confirm the mitochondrial localization of the Rhod-2 probe, cells were loaded with 1.0 μM Rhod-2 in HBSS for 30 min at 37 °C. The cells were then washed with HBSS and incubated with Hoechst 33342 to nuclei staining for 5 min, washed with HBSS, and visualized by the fluorescence microscopy using LEICA laser scan microscope.

### Immunofluorescence staining

After treatments, cells were loaded with Mito tracker (Cayman Chemical, Ann Arbor, MI, USA) in PBS for 30 min at 37 °C, washed with PBS and then the cells were fixed with methanol for 10 min at −20 °C and blocking in 5% BSA in PBST for 30 min. Fixed cells were incubated overnight at 4 °C with primary antibody nephrin (ab216341, Abcam, Cambridge, MA, USA), cytochrome c (12963S, Cell Signaling, Beverly, MA) or TRPC6 (ab62461, Abcam, Cambridge, MA, USA) diluted in PBST and then washed three times in PBST and incubated for 1 h at room temperature with anti-rabbit or anti-mouse Alexa Fluor 488 or 594 (A-11059 and A27027, Molecular Probes). The mitochondria were labeled with Mito tracker and the nuclei were visualized by DAPI (ab228549, Abcam, Cambridge, MA, USA) staining. Images were taken using LEICA laser scan microscope.

### Apoptosis assay by flow cytometry

Podocytes were plated in 12-well plates and cultured under the indicated conditions. Cell apoptosis was performed by FITC Annexin V Apoptosis Detection Kit (556547, BD Biosciences, Franklin Lakes, NJ, USA) following the manufacturer’s protocol. Cells were incubated with propidium iodide (PI) in the dark and were analyzed by flow cytometry on the FACSCalibur, using the CELLQuest software. Apoptotic podocytes were defined as annexin V-positive/PI-negative (early apoptotic) and annexin V-positive/PI-positive (late apoptotic) cells.

### Mitochondrial membrane potential staining

The mitochondrial membrane potential (MMP) was determined using a JC-1 assay kit (M34152, Invitrogen, San Diego, CA, USA). Treated cells were harvested, washed in ice-cold PBS, and stained with 2.5 μM JC-1 for 30 min at 37 °C, and the changes in MMP was analyzed by flow cytometry (BD FACSCalibur, Franklin Lakes, NJ, USA). The data were then analyzed with Flowjo7.6.1 software (FlowJo LLC, Ashland, OR, USA).

### Measurement of mitochondrial and cytosolic Ca^2+^ levels

To measure mitochondrial Ca^2+^, treated cells were incubated with 2.0 μM Rhod-2 in PBS at 37 °C for 30 min, washed with Krebs-ringer (without Ca^2+^) at 4 °C, and then analyzed by flow cytometry.

To measure cytosolic Ca^2+^, treated cells were incubated with 2.5 μM Fluo-4 at 37 °C for 30 min, washed with Krebs-ringer (without Ca^2+^), and analyzed immediately by flow cytometry.

### TRPC6 siRNA and transfection

The TRPC6-specific siRNA was synthesized and purchased from Genechem (Shanghai Co. Ltd.). siRNA transfection was carried out with Lipofectamine RNAiMAX Transfection Reagent (bothfromInvitrogen, Carlsbad, CA, USA) according to the manufacturer’s instruction. After 24 h plasmid transfection, podocytes were pretreated with or without 80 μM AS-IV for 12 h followed by incubation with BSA or 250 μM palmitate acid for 24 h and then harvested for further analysis.

### Preparation of cellular fractions and western blot analysis

Cytosolic and mitochondrial protein fractions were isolated using Cell Mitochondria Isolation Kit (89874, Thermo Fisher Scientific, Waltham, MA, USA) according to the instruction of manufacturer. The lysis buffer for isolating total cellular fractions contains 50 mm Tris (pH 7.4), 150 mm NaCl, 1% Nonidet P-40, 10% glycerol, and 5μl/ml of a mixture of protease inhibitors (Sigma-Aldrich, St. Louis, MO, USA). Protein concentrations were determined using the bicinchoninic acid protein assay kit (Beyotime Co, China). Proteins were separated by SDS-PAGE and western blotting was carried according to the standard procedures. Antibodies used were Bip (ab21685, Abcam, Cambridge, MA, USA), CHOP (ab11419, Abcam, Cambridge, MA, USA), cleaved caspase-3 (9664S, Cell Signaling, Beverly, MA), caspase-9 (9504S, Cell Signaling, Beverly, MA), Bax (5023, Cell Signaling, Beverly, MA), Bcl-2(15071, Cell Signaling, Beverly, MA), cytochrome c (Cell Signaling, Beverly, MA), TRPC6 (ab62461, Abcam, Cambridge, MA, USA), β-actin (4967S, Cell Signaling, Beverly, MA), β-tubulin (2146S, Cell Signaling, Beverly, MA) and VDAC (ABCA2271302, Epitomics, Burlingame, CA, USA). Complexes formed were detected with horseradish eroxidase-conjugated anti-mouse IgG or anti-rabbit IgG antibodies (Cell Signaling, Beverly, MA) using chemiluminescent reagents (Merck Millipore. Billerica, MA, USA). The blot images were produced by ImageQuant LAS 500 imaging system (GE Healthcare Bio-sciences AB, Uppsala, Sweden). Densitometry quantitation was performed using Image J 1.37 software (NIH, Bethesda, MD, USA). Protein expression was normalized with β-actin for total proteins, β-tubulin for cytosolic proteins and VDAC for mitochondrial proteins.

### Statistical analysis

Statistical analysis was conducted with GraphPad Prism software 5.0 software (GraphPad Software Inc., San Diego, CA, USA). One-way ANOVA followed by the Newman-Keuls multiple comparisons test was used for statistical comparisons among experimental groups, with a value of *P* < 0.05 being considered statistically significant. Data are expressed as the means ± SEM.

## Results

### AS-IV inhibited PA-induced ER stress and podocyte apoptosis

**Fig. 1:**
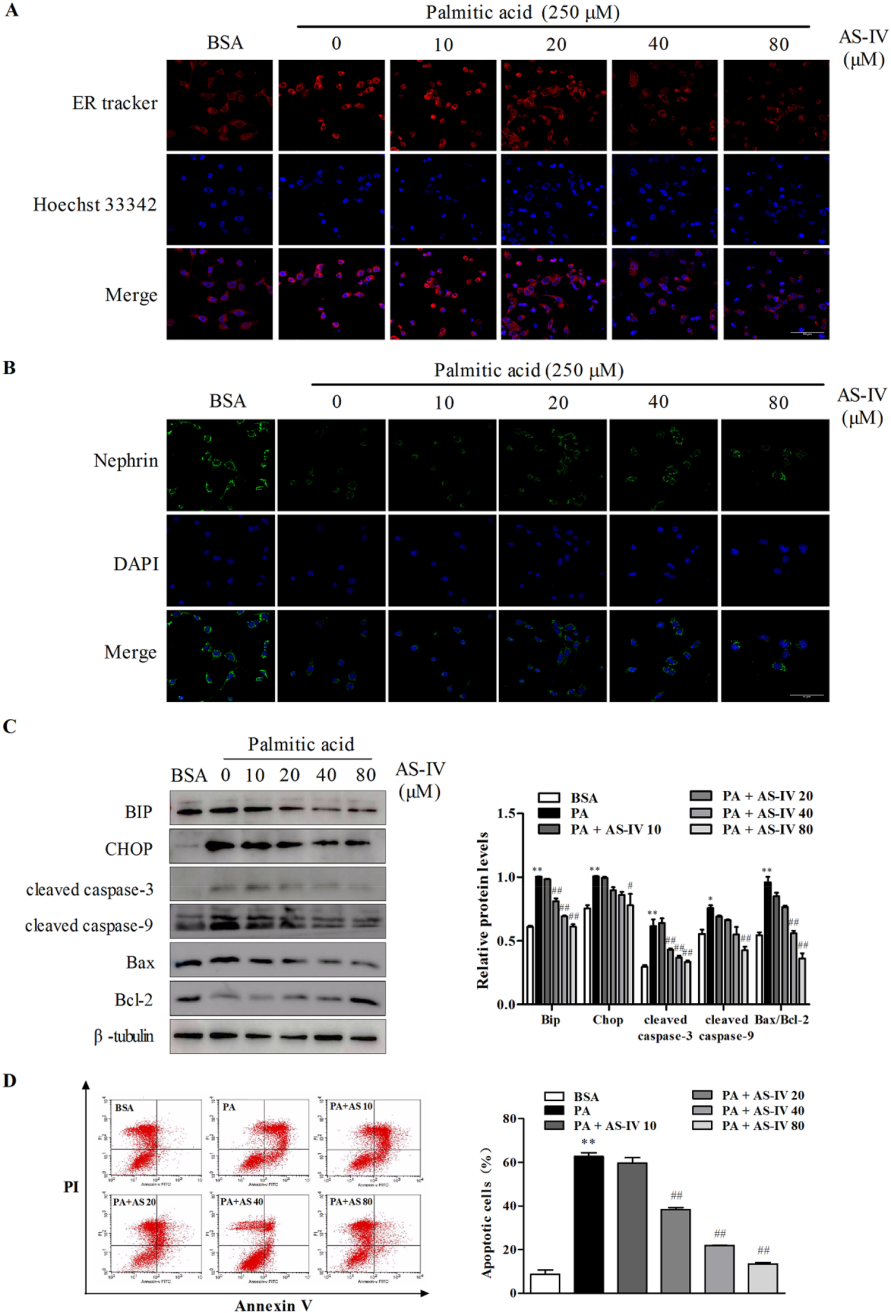
Effect of AS-IV on palmitic acid (PA)-induced ER stress and podocyte apoptosis. Podocytes were pretreated with or without AS-IV at the indicated concentrations (0, 10, 20, 40 and 80 μM, respectively) for 12 h followed by 250 μM palmitate exposure for 24 h. (A-B) Representative confocal microscopic images of (**a**) ER-tracker and (**b**) nephrin in podocytes with different cultural treatment. Scale bars, 10 μm; Original magnification, ×400. **c** Representative immunoblots and densitometry quantification of BIP, CHOP, cleaved caspase 3, 9, Bax and Bcl-2 expression in podocyte with different cultural treatment. **d** Representative flow cytometry images and quantitative analysis of apoptotic podocytes with different cultural treatment. Data are presented as means ± SEM. n = 3 for each group (**a**–**d**). *p < 0.05, **p < 0.01, compared with BSA-treated podocyte; ## p < 0.01, compared with PA-treated podocyte

To evaluate the anti-apoptosis activity of AS-IV, podocytes were exposed to palmitic acid (PA) and AS-IV at the indicated concentrations. As shown in Fig. 1, with PA treatment endoplasmic reticulum (ER) structure co-stained with ER tracker and Hoechst 33342 revealed an increased ER tracker fluorescence, while the staining assay of Nephrin, a classical podocyte foot process marker, showed a decreased change (Fig. 1a, b). ER contains a pool of molecular chaperones including BiP, the latter was identified as one of the representative markers which is induced in the process of ER stress. Then the apoptotic signaling will be activated through downstream molecules including CHOP. In Fig. 1c, western blot analysis confirmed Bip and CHOP expression increased. And the following apoptosis signaling molecules, such as the pro-apoptotic (CHOP, Bax) transcription factors also increased while the antiapoptotic (Bcl-2) transcription factors decreased with PA treatment. Up-regulation of Bax/Bcl-2 ratio means promotion of apoptosis occurred. Moreover, these results were further supported by the number of apoptosis cells detected by flow cytometry (Fig. 1d). In contrast, addition of AS-IV exhibited conversely effect in a dose-dependent manner opposite to PA treatment, which indicating AS-IV may protect against PA-induced ER stress and podocyte apoptosis.

### AS-IV inhibited cytochrome c release and decreased mitochondrial transmembrane potential

**Fig. 2:**
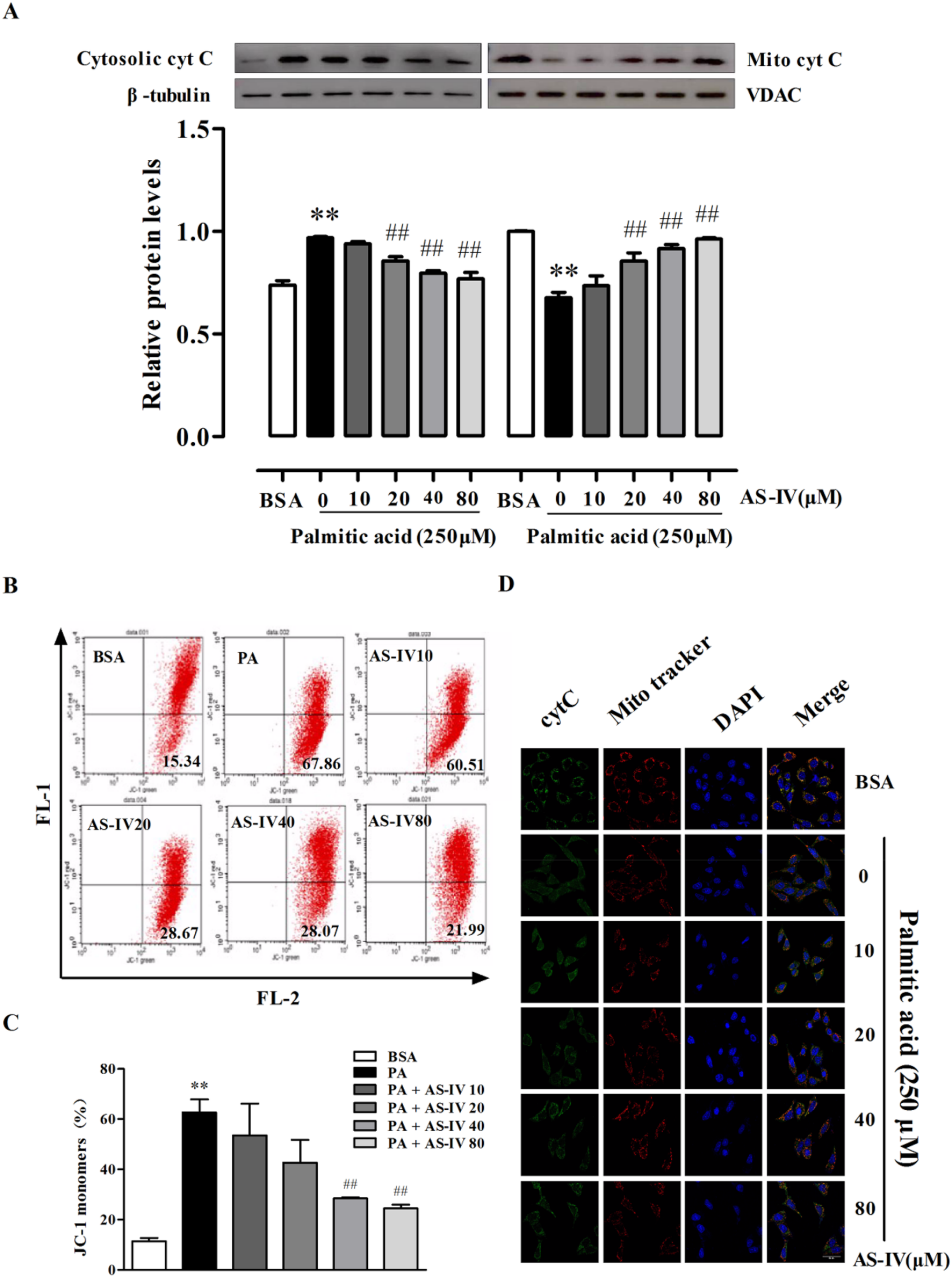
Effect of AS-IV on PA-induced cytochrome c release and mitochondrial transmembrane potential. **a** Representative immunoblots and densitometry quantification of translocation of cytochrome c in podocytes with different cultural treatment. **b**–**c** Representative images analyzed by JC-1 (**b**) and quantitative analysis (**c**) of MMP changes in podocytes with different cultural treatment. (D) Representative confocal microscopic images of cytochrome c and Mito tracker coexpression in podocytes with different cultural treatment. Scale bars, 10 μm; Original magnification, **×** 400. Data are presented as means ± SEM. n = 3 (**a**, **b**) and n = 5 (**d**) for each group. **p < 0.01, compared with BSA-treated podocyte; ##p < 0.01, compared with PA-treated podocyte

Mitochondria is a vital organelle that regulating cellular fate including apoptosis. Cleaved caspase-3, 9 expressions in western blot suggested the involvement of mitochondria-related apoptosis signaling in AS-IV effect to podocyte (Fig. 1c). In Fig. 2a–c, PA significant induced mitochondrial dysfunction which existed reflected by the increased efflux of cytochrome c into cytosol and the elevated changes of mitochondrial transmembrane potential (p < 0.01). Conversely, AS-IV dose-dependently decreased the cytochrome c efflux and mitochondrial transmembrane potential (p < 0.01). Co-stained with cytochrome c and Mito tracker also confirmed the beneficial effect of AS-IV to podocyte (Fig. 2d).

### AS-IV regulated endoplasmic reticulum, mitochondrial and cytosolic Ca^2+^ levels

**Fig. 3:**
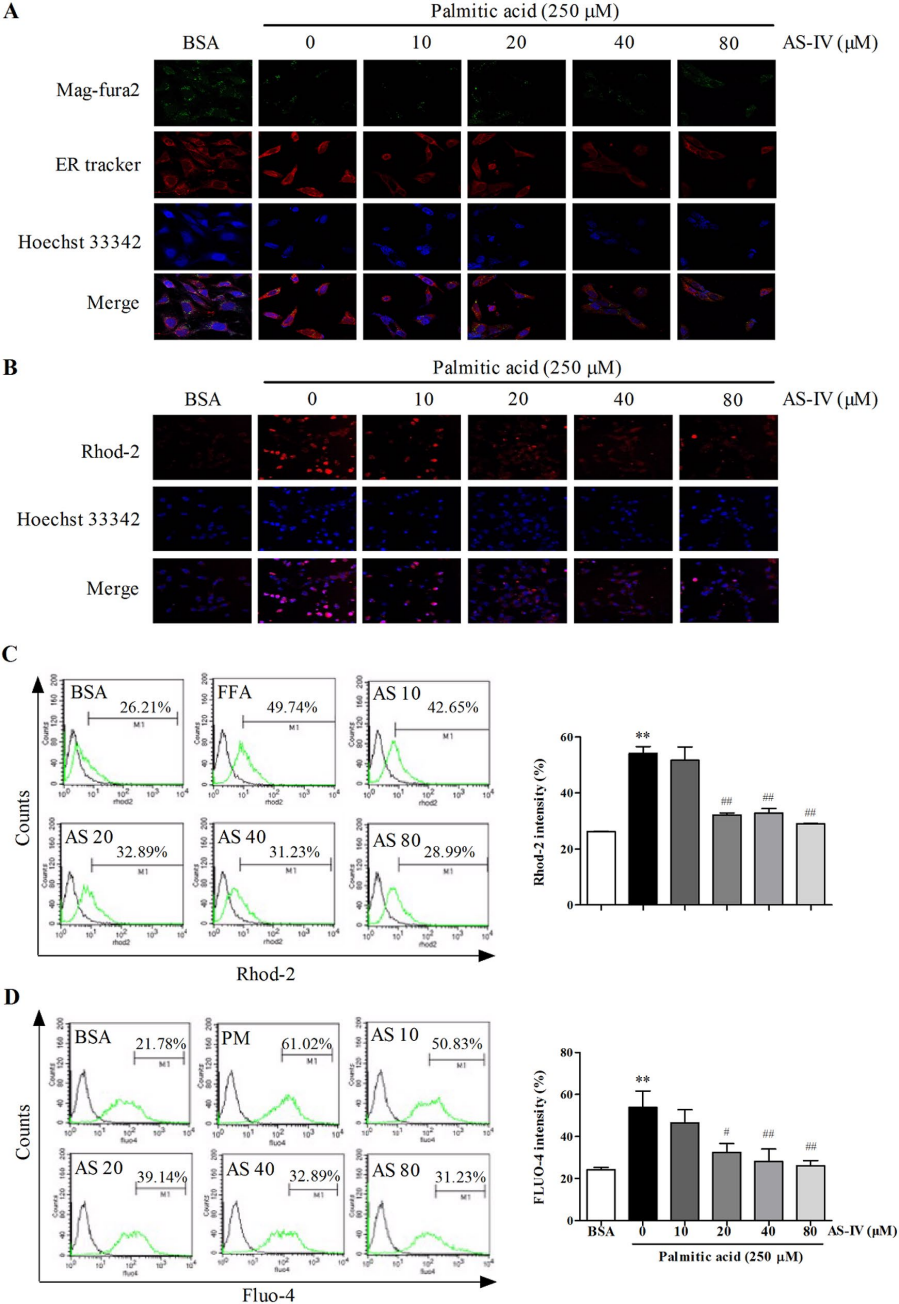
Effect of AS-IV on endoplasmic reticulum, mitochondrial and cytosolic Ca^2+^ levels. (**a**–**b**) Representative confocal microscopic images of (**a**) Mag-fura2, ER-tracker and (**b**) Rhod-2 in podocytes with different cultural treatment. Scale bars, 10 μm; Original magnification, **×** 400. **c** Representative images analyzed by flow cytometric and quantitative analysis of Rhod-2 in podocytes with different cultural treatment. **d** Representative pictures analyzed by flow cytometric and quantitative analysis of Fluo-4 in podocytes with different cultural treatment. Data are presented as means ± SEM. n = 3 for each group (**a**–**d**). **p < 0.01, compared with BSA-treated podocyte; #p < 0.05, ##p < 0.01, compared with PA-treated podocyte

PA impacts podocytes fate via regulating cytosolic Ca^2+^ flux, the latter correlate with the levels of ER and mitochondrial Ca^2+^ levels. As shown in Fig. 3a, cells were treated with Mag-fura2, a low-affinity Ca^2+^ dye known to localize to the endoplasmic reticular lumen, co-localized with ER-tracker and Hoechst 33342 in podocytes. Meanwhile, Rhod-2 was also used to monitor mitochondrial Ca^2+^ concentration in Fig. 3b, c. In addition, Fluo-4 staining assay was performed by flow cytometry to detect the cytosolic Ca^2+^ levels of podocytes (Fig. 3d). The results showed that with PA treatment, ER Ca^2+^ depletion existed while mitochondrial and cytosolic Ca^2+^ levels increased (p < 0.01). Accordingly, AS-IV seemed to reverse the disturbance in a dose-dependent manner, especially significant at 20, 40 and 80 μM (p < 0.05).

### AS-IV inhibited PA-induced podocyte apoptosis is Ca^2+^ dependent

**Fig. 4:**
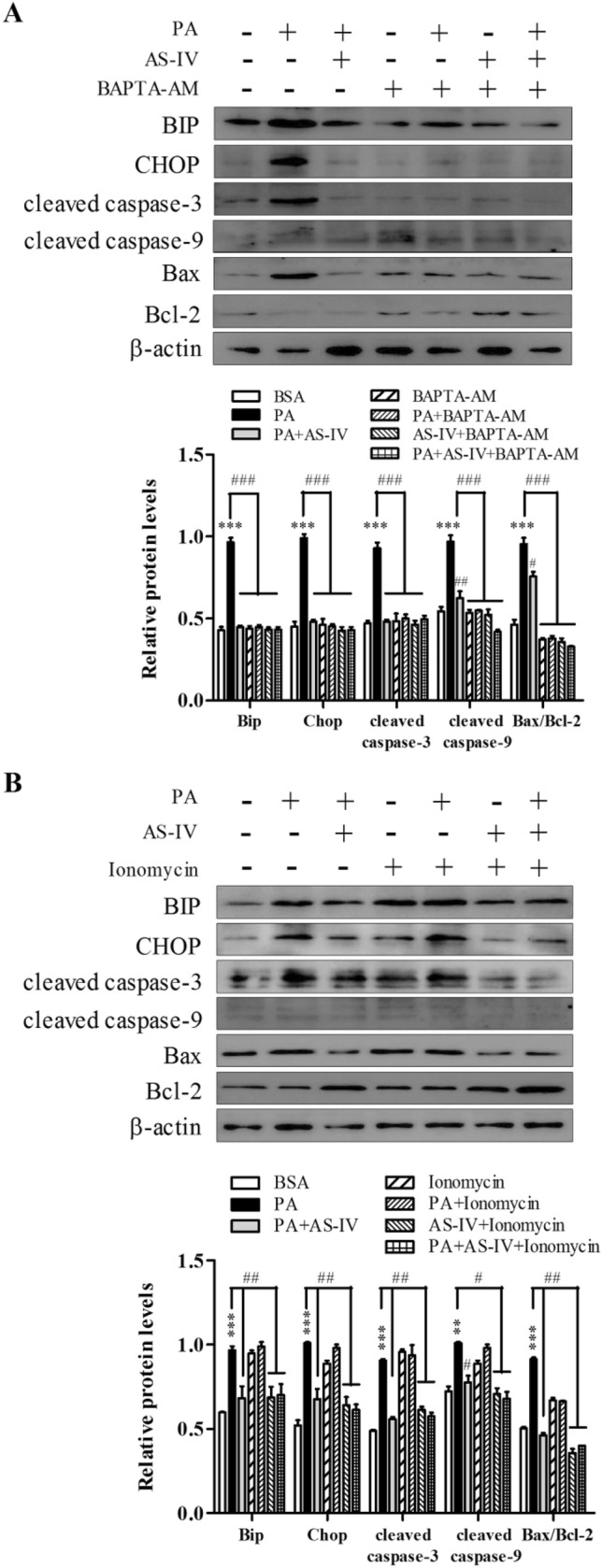
Effect of Ca^2+^ to efficacy of AS-IV in suppressing PA-induced podocyte apoptosis. Podocytes were either untreated or pretreated with 80 μM AS-IV for 12 h before addition of either in the presence 10 μM BAPTA-AM (**a**) or 5 μM ionomycin (**b**) for 1h as indicated. Treated cells were immunoblotted with antibodies to Bip, CHOP, cleaved caspase 3,9, Bax and Bcl-2. Data are presented as means ± SEM. n = 3 (**a**, **b**) for each group. **p < 0.01, ***p < 0.001, compared with BSA-treated podocyte; ##p < 0.01, ###p < 0.001, compared with PA-treated podocyte

To further test the possible role of Ca^2+^ in the regulated effect of AS-IV to podocyte apoptosis, BAPTA-AM and ionomycin were used to monitor the process of apoptosis. As shown in Fig. 4a, in the presence of BAPTA-AM, a cell-permeable acetoxymethyl ester of the Ca^2+^ scavenger BAPTA, PA-induced Bip, CHOP, cleaved caspase-3,9 and Bax/Bcl-2 expression was significantly suppressed (p < 0.01). Even with treatment of AS-IV the inhibited efficacy was no more relieved. Contrarily, ionomycin, an ionophore of Ca^2+^, increased the three proteins expression significantly both in the presence and absence of PA (p < 0.001). However, AS-IV treatment ameliorated the increased tendency (p < 0.01). Thus, the opposite efficacy of BAPTA-AM and ionomycin here confirmed Ca^2+^ take part in AS-IV effect to inhibit PA-induced podocyte apoptosis.

### TRPC6 involved in the process of AS-IV to suppress PA-induced podocyte apoptosis

**Fig. 5:**
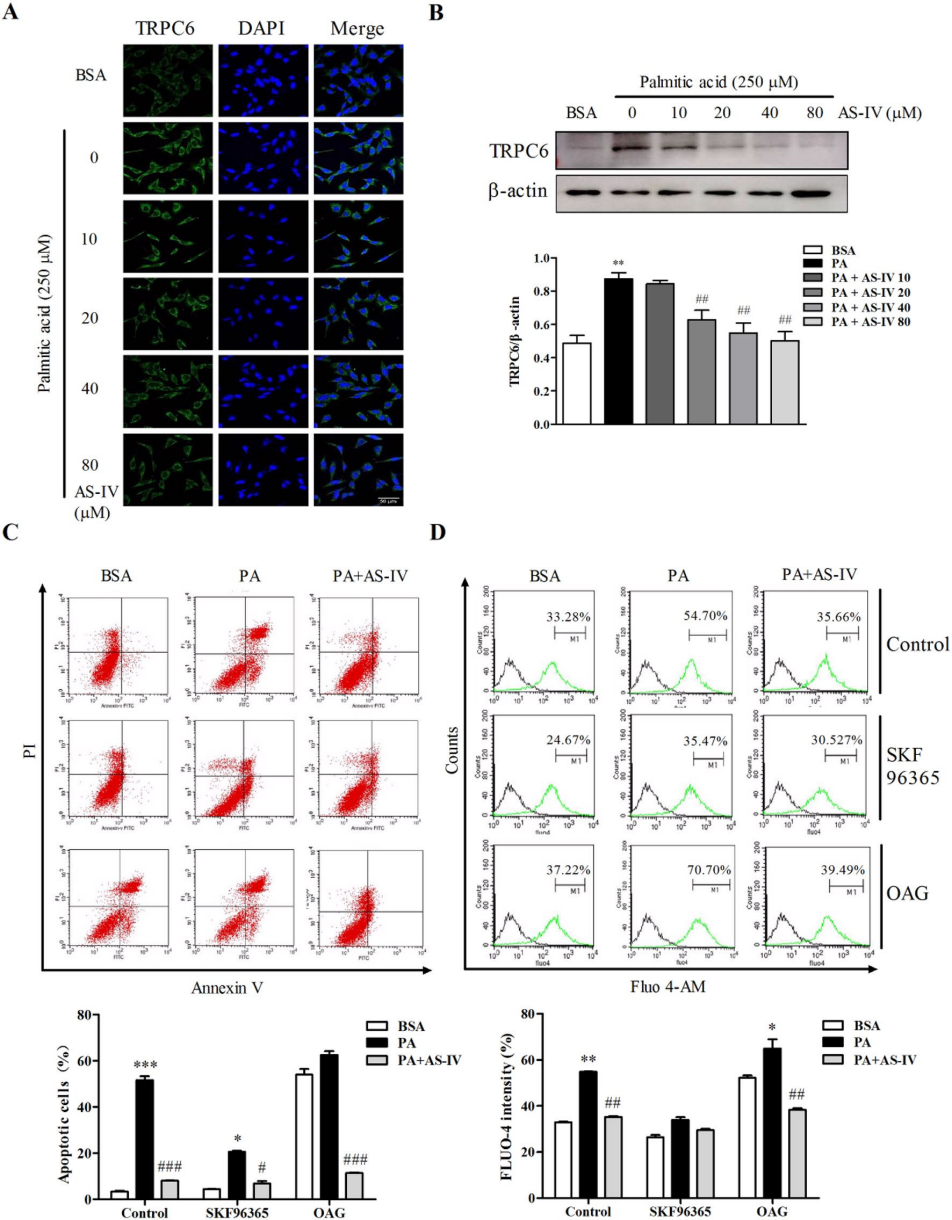
Effect of TRPC6 to AS-IV inhibited PA-induced podocyte apoptosis. Podocytes were pretreated with or without AS-IV at the indicated concentrations (0, 10, 20, 40 and 80 μM, respectively) for 12h followed by 250 μM palmitate acid exposure for 24 h. **a** Representative confocal microscopic images of TRPC6 in podocytes with different cultural treatment. Scale bars, 50 μm; Original magnification, ×400. **b** Representative immunoblots and densitometry quantification of TRPC6 expression in podocyte with different cultural treatment. Before podocytes were treated with BSA, PA and 80 μM AS-IV as indicated, the cells were incubated with either in the presence 10 μM SKF96365 or 20 μM OAG for 1h for (**c**) representative flow cytometry pictures and quantitative analysis of apoptotic podocytes with different cultural treatment and (**d**) representative images analyzed by flow cytometric and quantitative analysis of Fluo-4 in podocytes with different cultural treatment. Data are presented as means ± SEM. n = 5 (**a**) and n = 3 (**b**–**d**) for each group. *p < 0.05, **p < 0.01, ***p < 0.001, compared with BSA-treated podocyte; #p < 0.05, ##p < 0.01, ###p < 0.001, compared with PA-treated podocyte

TRPC6-mediated calcium influx is considered correlate with podocyte apoptosis. In Fig. 5a–b, both TRPC6 staining and protein expression increased with PA treatment (p < 0.01), while AS-IV decreased the expression in a dose-dependent manner, especially significant at 20, 40 and 80 μM (p < 0.01). When the podocyte exposed to SKF96365, an antagonist of TRPC6, PA-induced apoptosis and cytosolic Ca^2+^ level was depressed while AS-IV reversed the effect significantly (p < 0.01). However, the agonist of TRPC6 (OAG) exhibited adverse efficacy. To further identify the involvement of TRPC6 in calcium regulation, TRPC6-specific siRNA was introduced. As shown in Supplementary Figure, the increased expression of Bip, CHOP, cleaved caspase-3,9 and Bax/Bcl-2 were decreased with 80 μM AS-IV treatment in control siRNA. However, the inhibitory of AS-IV to the three proteins were abolished in TRPC6 siRNA. Furthermore, apoptosis assay and Fluo-4 staining assay detected by flow cytometry also showed the no significant role of AS-IV in TRPC6 siRNA. Thus, these results indicate TRPC6 may be the potential target for AS-IV to inhibit PA-induced podocyte apoptosis.

## Discussion

Podocytes, which are visceral epithelial cells located at the outer aspect of the capillary loops, play a prominent role in maintaining the integrity of the glomerular filter [2]. *Astragalus membranaceus (Fisch) Bge* is one of the frequently prescribed for renal disorders in traditional Chinese medicine and has been made as tablets for convenient application. As the main bioactive component of *Astragalus membranaceus (Fisch) Bge*, AS-IV could be used clinically for the treatment of diabetes as for the comprehensive pharmacological actions which have been reported both in vitro and in vivo experiments [15]. Previous study has shown that AS-IV at 50, 100, 200, 400 and 800 μg/mL is benificial to against podocyte injury, the result is consistent with our another studies in which AS-IV below 80 μmol (equal to 62.8 μg/mL) can ameliorate PA- and high glucose-induced podocyte apoptosis based on apoptosis assay using flow cytometry and other in vitro experiments [13, 14, 16]. So in this study, AS-IV concentration at 20, 40, 80 μmol were continue used to examine the antiapoptotic effect of this component. This study provides new evidence showing that AS-IV participates in abnormal Ca^2+^ homeostasis in PA-stimulate podocyte apoptosis. The process correlated with inhibited Ca^2+^ release and Ca^2+^ influx, and evoked Ca^2+^-triggered apoptosis. Insights into podocyte apoptosis from regulating Ca^2+^ would provide a better understanding of DN pathogenesis and thus help develop AS-IV as a promising adequate agent in therapeutic process.

Normally, intracellular Ca^2+^ homeostasis is vital in the control of cellular processes and Ca^2+^ overload is known to initiate processes leading to cell death [17]. Recently the irreplaceable action of Ca^2+^ dynamics in physiological podocyte functions and proteinuria kidney diseases including DN has been highlighted [18, 19]. Disrupt intracellular Ca^2+^ homeostasis can increase Ca^2+^ release from intracellular Ca^2+^ store, or through reducing the influx of extracellular Ca^2+^. Here we found that scavenging of intracellular Ca^2+^ using BAPTA-AM significantly rescued podocyte apoptosis from PA injury, while ionomycin exerted opposite modulation (Fig. 4). These pharmacological interventions demonstrate the functional importance of intracellular Ca^2+^ levels to podocyte function. ER is the major intracellular store of Ca^2+^, increased cytosolic Ca^2+^ from ER calcium depletion contribute to β–cell death and identification of ideal compounds restoring ER Ca^2+^ levels is a novel therapeutic modalities for type 1 and type 2 diabetes [20]. Additionally, continuous evidences have elucidated that ER stress accompanied with the Ca^2+^ regulation in ER and correlate with PA-induced podocyte apoptosis [21–23]. In this context, we presented here that PA can induce Ca^2+^ release from ER store and lead to podocyte ER stress, as evidenced by upregulation of the Bip and CHOP expression. Meanwhile, we found that AS-IV downregulated Bip and CHOP expression, increased ER Ca^2+^ localization and ameliorated cytosolic Ca^2+^ level in dose-dependence (Fig. 1). Those data suggest the contribution of AS-IV to inhibit podocyte apoptosis may correlate with regulation of Ca^2+^ release from ER.

Besides ER, mitochondrion is another well-known major reservoir of intracellular Ca^2+^. Mitochondrial Ca^2+^ uptake contributes to cytosolic Ca^2+^ shaping thus impinging on specific Ca^2+^-dependent events [24]. The regulation of mitochondrial Ca^2+^ concentration includes the balance between influx and efflux pathways, including the pro-uptake activity of the mitochondrial Ca^2+^ uniporter, the pro-release activity of sodium-calcium exchanger, sodium-hydrogen exchanger and mitochondrial permeability transition pore [25]. Mitochondrial dysfunction is considered to be closely associated with the regulation of cell death, including podocyte apoptosis [26, 27]. In this study, as anticipated we found that the expression of cleaved caspase-3,9 translocation of cytochrome c and mitochondrial membrane potential increased with PA treatment. Along with this, mitochondrial Ca^2+^ level is also raised. Whereas PA-induced apoptosis could be inhibited by AS-IV through the suppression of the mitochondrial release of cytochrome c to the cytosol, the attenuation of mitochondrial membrane potential and the reversal of PA-activation of cleaved caspase-3,9 (Fig. 2). Thus, our data confirmed that PA can induce elevation of intracellular Ca^2+^ level and trigger mitochondrial damage while AS-IV takes adverse effect. Our previous study revealed that Ca^2+^ uptake via mitochondrial uniporter contributes to PA-induced apoptosis in mouse podocytes, which independent of IP_3_R and RyR in ER [5]. In this study, we indeed found that the expression of mitochondrial uniporter increased with PA treatment and AS-IV can decreased the expression dose-dependently (data not shown). And all these results, at least in part, indicate that as well as ER, mitochondrial Ca^2+^ also participated in the signaling of AS-IV to attenuate PA-induced podocyte apoptosis. These findings are consistent with previous studies that ER and mitochondria can interact both physiologically and functionally, especially at Ca^2+^ regulation [28–30]. The process is mediated by protein misfolding within the ER, which results in release of Ca^2+^ from the intracellular stores into the cytosol, then Ca^2+^ released from ER is taken up by mitochondria and results in Ca^2+^ overload and induces depolarization of permeability transition pore and cell death [31]. Therefore, we speculate that the stimulation of PA trigger release of ER Ca^2+^ may contributes to further ER stress, evidenced by the accumulation of misfolded proteins in ER, as our results supported. In addition, the possibility that mitochondrial uptake of Ca^2+^ may exist with the following process and result to the ultimate podocyte apoptosis. According, it is assumed that ER and mitochondria cooperate to signal PA-induced apoptosis via Ca^2+^ and AS-IV take part in the crosstalk interference, the molecular mechanism warrants further investigation.

Stimulation of the calcium-sensing receptor constitutes a new approach to stabilize podocyte cytoskeleton, improves cell survival, and reduces glomerulosclerosis [32]. As extracellular Ca^2+^ originate, TRPC channels have been proposed as store-operated calcium channel candidates [33]. Notably, TRPC6 is a confirmed channel which located on podocyte slit diaphragms and is sensitive to Ca^2+^ overload [34, 35]. In this study, we found that cytosolic Ca^2+^ increased significantly induced by PA, it is reasonable to propose the elevation of intracellular Ca^2+^ may come from TRPC6. Meanwhile, treatment with AS-IV decreased the elevation, so the direct target of AS-IV is TRPC6. From the present data, AS-IV can depress the expression of TRPC6, accompany with the decrease of intracellular Ca^2+^, ER stress-associated proteins and apoptotic cells. Increased expression of TRPC6 with OAG can significantly increase the influx of Ca^2+^ into cytosolic in the context of PA introduction. Conversely, the rise of Ca^2+^ did not occur in TRPC6 inhibition and siRNA (Fig. 5 and supplementary Figure). The results support TRPC6 involves in PA-induced calcium influx to promote podocyte apoptosis associated with ER stress. That is to say TRPC6 is the downstream of PA and is the target of AS-IV. This finding consistent with another study in which AS-IV may ameliorate high-glucose induced podocyte apoptosis relating with the down-regulation of TRPC6 [36]. These findings highlight the crucial role of Ca^2+^ in regulating podocyte apoptosis and TRPC6 may be the source of Ca^2+^. We can see here the protein expression of TRPC6 is really depressed by AS-IV, but whether it acting as a channel inhibitor still needs further investigation. Beyond that, we noticed that SKF96365 could not counteract the pro-apoptosis of PA completely, even the beneficial efficacy of AS-IV. We proposed that TRPC6 may not the only target to AS-IV, in other words, beyond just TRPC6 some other ion channel or receptor might also involve in control Ca^2+^ influx from extracellular milieu. Notably, other TRPC channels can really exert regulation in podocyte apoptosis [37, 38].

In summary, we have shown that PA-induced ER stress and following apoptosis is correlate with intracellular Ca^2+^ level disturbance, relating to the abnormal flow of Ca^2+^ in ER and mitochondria, and TRPC6 may also partly involved in the Ca^2+^ influx from extracellular milieu. In contrast, AS-IV exerted inhibitory efficacy to regulate those disturbance in a dose-dependent manner. Thus, our study provides an encouraging example of the therapeutic potential of AS-IV in modulating podocyte apoptosis and more studies are needed before AS-IV could be used as a therapeutic agent.

## Supplementary Information

Below is the link to the electronic supplementary material.

Supplementary Figure Effect of TRPC6 siRNA to AS-IV inhibited PA-induced podocyte apoptosis. Podocytes were transfected with control siRNA and TRPC6 siRNA plasmids for 24h and treated with or without AS-IV at 80 μM for 12h followed by 250 μM palmitate acid exposure for 24 h. a Representative immunoblots and densitometry quantification of TRPC6 expression transfected with control siRNA and TRPC6 siRNA plasmids in podocyte. b Representative immunoblots and densitometry quantification of BIP, CHOP, cleaved caspase 3,9, Bax and Bcl-2 expression in podocyte with different cultural treatment. c–d Representative flow cytometry images and quantitative analysis of apoptotic podocytes (c) and Fluo-4 (d) in podocyte with different cultural treatment. Data are presented as means ± SEM. n = 3 (A-D) for each group. *p < 0.05, **p < 0.01, ***p < 0.001, compared with BSA-treated podocyte; ## p < 0.01, ### p < 0.001, compared with PA-treated podocyte (DOCX 758 KB)
