# Supplementary figures and images for: Astragaloside IV inhibits palmitic acid-induced apoptosis through regulation of calcium homeostasis in mice podocytes
Source: Mol Biol Rep. 2021 Feb 19;48(2):1453–64. doi: 10.1007/s11033-021-06204-4 (PMC7925475; doi:10.1007/s11033-021-06204-4)

**Supplementary Figure**.


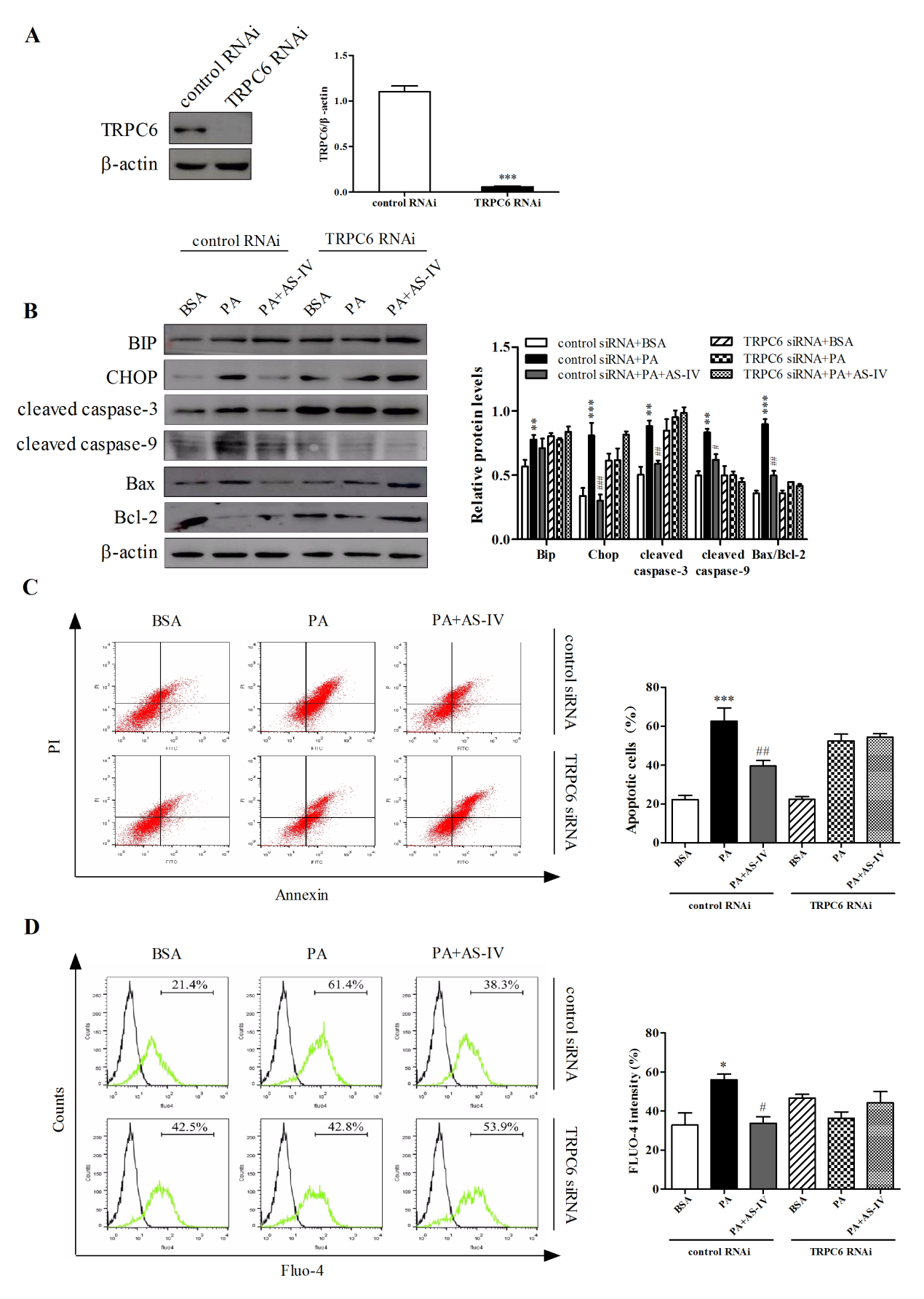

Supplement: Supplementary file 1 — Supplementary Figure Effect of TRPC6 siRNA to AS-IV inhibited PA-induced podocyte apoptosis. Podocytes were transfected with control siRNA and TRPC6 siRNA plasmids for 24h and treated with or without AS-IV at 80 μM for 12h followed by 250 μM palmitate acid exposure for 24 h. a Representative immunoblots and densitometry quantification of TRPC6 expression transfected with control siRNA and TRPC6 siRNA plasmids in podocyte. b Representative immunoblots and densitometry quantification of BIP, CHOP, cleaved caspase 3,9, Bax and Bcl-2 expression in podocyte with different cultural treatment. c–d Representative flow cytometry images and quantitative analysis of apoptotic podocytes (c) and Fluo-4 (d) in podocyte with different cultural treatment. Data are presented as means ± SEM. n = 3 (A-D) for each group. *p < 0.05, **p < 0.01, ***p < 0.001, compared with BSA-treated podocyte; ## p < 0.01, ### p < 0.001, compared with PA-treated podocyte (DOCX 758 KB) [file 11033_2021_6204_MOESM1_ESM.docx]
